# The formation of SCEs as an effect of occupational exposure to formaldehyde

**DOI:** 10.1007/s00204-022-03238-w

**Published:** 2022-02-12

**Authors:** Federica Ghelli, Enrico Cocchi, Valeria Bellisario, Martina Buglisi, Giulia Squillacioti, Alfredo Santovito, Roberto Bono

**Affiliations:** 1 Department of Public Health and Pediatrics, University of Turin, Via Santena 5 bis, 10126 Turin, Italy; 2 Department of Life Sciences and Systems Biology, University of Turin, Via Accademia Albertina 13, 10123 Turin, Italy

**Keywords:** Formaldehyde, Genotoxicity, Biomonitoring, Occupational exposure

## Abstract

Formaldehyde (FA) is a ubiquitous toxic chemical employed worldwide due to its disinfectant and preservative properties. Despite being classified as a human carcinogen, FA is still employed as formalin in pathology wards as standard fixative. We evaluated its relationship with the formation of sister-chromatid exchanges (SCEs) in cultured peripheral blood lymphocytes on 57 pathologists and 48 controls and the risk/protective role played by several genetic polymorphisms. All subjects were assessed for SCEs and genotyped for the most common cancer-associated gene polymorphisms: *CYP1A1* exon 7 (A > G), CYP1A1*2A (T > C), CYP2C19*2 (G > A), *GSTT1* (presence/absence), *GSTM1* (presence/absence), *GSTP1* (A > G), *XRCC1* (G399A), *XRCC1* (C194T), *XRCC1* (A280G), *XPC* exon 15 (A939C), *XPC* exon 9 (C499T), *TNFα* − 308 G > A), *IL10* − 1082 (G > A), and *IL6* − 174 (G > C). Air-FA concentration was assessed through passive personal samplers. Pathologists, exposed to 55.2 μg/m^3^ of air-FA, showed a significantly higher SCEs frequency than controls, exposed, respectively, to 18.4 μg/m^3^. Air-FA was directly correlated with SCEs frequency and inversely with the replication index (RI). Regression models showed FA exposure as a significant predictor in developing SCEs, while did not highlight any role of the selected polymorphisms. Our study confirms the role of low air-FA levels as genotoxicity inductor, highlighting the importance to define exposure limits that could be safer for exposed workers.

**Supplementary Information:**

The online version contains supplementary material available at 10.1007/s00204-022-03238-w.

## Introduction

Formaldehyde (FA) is a ubiquitous toxic highly reactive chemical with carcinogenic properties (Zhao et al. 2021; Kang et al. 2021). FA is the result of both natural and anthropogenic processes, and its industrial employment is extended worldwide. It is a well-known disinfectant and preservative to which humans are exposed mainly by respiratory way, in both life and occupational settings. Workers directly involved in the production or use of FA and FA-based compounds, are exposed to higher concentration of this pollutant than the general population (Motta et al. 2021; Kang et al. 2021). Due to its ability in preserving cell and tissue morphology, formalin, an aqueous solution containing dissolved FA, is widely used in pathology wards (Shaham et al. 2002; Motta et al. 2021). Pathologists are exposed to FA in several steps of their workflow. The most relevant exposure happens in the grossing room, when workers manipulate the anatomic samples soaked in formalin. Particularly, “sampling” step, when pathologists handle the pieces from formalin container and the “rinsing” step to avoid the formalin excess under running water before slicing are the occasion when the proximity between the operator and the formalin jar determine the higher exposure to FA vapour (Vimercati et al. 2010). FA is currently classified as human carcinogen, based on studies on nasopharyngeal cancer and leukaemia (IARC Working Group on the Evaluation of Carcinogenic Risks to Humans. et al. 2006). The mechanisms by which FA could be involved in causing cancer are not completely clear to date; several pathways have been proposed, including epigenetic effects, DNA reactivity, chromosomal breakage, and oxidative stress (Costa et al. 2015).

Cytogenetic biomarkers as Sister-Chromatid Exchanges (SCEs), assessed in cultured peripheral blood lymphocytes (PBL), are reliable tools widely employed in human biomonitoring to assess FA-related DNA damage level and, in turn, genotoxic effects (Norppa 2004; Santovito et al. 2014, 2015; Costa et al. 2019). SCEs are the result of DNA replication product interchanges between sister chromatids at apparently homologous *loci* (Norppa 2004). This phenomenon is due to molecules able to form covalent adducts to DNA or to interfere with DNA metabolic processes (Santovito et al. 2014).

Several studies to date assessed increased SCEs frequency as a consequence of FA exposure, both in vitro and in exposed worker’s PBL (Shaham et al. 2002; Speit et al. 2007; Costa et al. 2008, 2019; Bernardini et al. 2020).

In this scenario, the individual genetic background is a key factor in shaping the response to FA exposure (Bernardini et al. 2020), with several polymorphisms able to influence its genotoxic effect, that can be used as susceptibility biomarkers (Šrám and Binková 2000). As example, any polymorphism involved in the xenobiotic metabolism or in the DNA damage repair pathways may affect the individual susceptibility to genotoxic agents (Norppa 2004).

Enzymes involved in xenobiotic biotransformation are generally classified into Phase I and Phase II enzymes, according to the reactions they catalyse (Jancova et al. 2010). Phase I enzymes include mostly enzymes belonging to the cytochrome P (CYP) 450 (CYP450) family, which is a key factor in oxidative reactions, resulting in transformation of a parent compound to more polar metabolite(s) (Swinney et al. 2006; Jancova et al. 2010). The metabolites obtained are often electrophilic molecules able to bind proteins and nucleic acids, leading thus to an alteration of cellular functions (Aladesanmi et al. 2017). Conversely, phase II enzymes are mostly transferases responsible of conjugation reaction, aiming at the transformation of xenobiotic or phase I enzyme products into easily excretable molecules (Swinney et al. 2006; Jancova et al. 2010). One of its major components are glutathione S-transferases (GSTs), which catalyse the conjugation of the glutathione reduced form (GSH) (Tan et al. 2017). In this regard, subjects exposed to FA carrying the *CYP2E1* intronic polymorphism (rs6413432) seems to have higher levels of genetic damage (Costa et al. 2015). Similarly, *GSTM1* and *GSTT1* null-alleles seem to be related to an enhanced genotoxic effect in FA-exposed workers (Costa et al. 2008; Ghelli et al. 2021). On the other side, DNA repair mechanisms are crucial to counteract DNA damage due to xenobiotic and carcinogens exposure. Polymorphisms in genes involved in the Nucleotide Excision Repair (NER) and in the Base Excision Repair (BER) pathways have been associated with increased risk for several types of cancer, including head, neck, and lung cancer (Costa et al. 2008). Finally, FA is known to be a powerful oxidative stress (OS) inductor (Bellisario et al. 2016; Bono et al. 2016). OS and inflammation are interdependent processes leading to an alteration in the ROS production and, in turn, in the release of proinflammatory cytokines (Hussain et al. 2016). Cytokines are soluble factors involved in regulating and promoting the immune response at cellular level with a pleiotropic function (Santovito et al. 2012; Barnes and Somerville 2020). Polymorphisms in *IL-6* and *TGF-β*_*1*_ gene, coding, respectively, for a pro-inflammatory and an anti-inflammatory cytokine, seems to influence SCEs level in cultured human PBL of FA exposed workers (Santovito et al. 2016).

The possible role of phase I, phase II, DNA-repair, and cytokine gene polymorphisms in modulating the genotoxic effect of FA exposure in workers chronically exposed has to be better understand. Thus, we assessed the SCEs frequency in PBL of professionals working in pathology wards and genotyped them for a battery of gene polymorphisms. These last are: Cytochrome P450 1A1 (*CYP1A1*) exon 7 (A > G), *CYP1A1 2A* (T > C), CYP2C19*2 (G > A), *GSTT1*, *GSTM1*, *GSTP*, X-ray repair cross-complementing group 1 (*XRCC1*) 399 (G > A), 194 (C > T), 280 (A > G), *Xeroderma pigmentosum* complementation group C (*XPC*) exon 15 (A > C), exon 9 (C > T), *TNF-α, IL-6, IL-10* − 1082 (G > A).

## Materials and methods

### Epidemiological sample and questionnaire

The epidemiological sample for this study included 105 workers, 57 pathologists recruited in two pathological wards of two Turin hospitals (Northwestern Italy) and 48 healthcare workers, recruited in the same medical facilities. The control group included mainly nurses and support personnel operating in different wards and not involved in tasks requiring the manipulation or the exposure to FA-based chemicals, such as formalin, or other carcinogenic compounds. Each subject accepted voluntarily to participate and signed an informed consent form prior to their inclusion in the study. The study was approved by the University of Turin bioethical committee (Ethics Committee of Azienda Ospedaliera Città della Salute e della Scienza of Torino—protocol code 0071900, 25 June 2013 and protocol code 0094007, 9 May 2013) and was performed in accordance with ethical standards laid down in the 1964 Declaration of Helsinki and its later amendments. All the subjects working since at least 1 year who agreed to participate were enrolled.

Each subject enrolled, both belonging to the pathologists’ and the controls’ group, filled out a questionnaire, provided a blood specimen, and wore for an 8-h working shift an air-FA diffusive personal sampler.

An interviewer administered a questionnaire aiming to collect information about individual characteristics (sex, age), habits referring to the last year (smoking), and working characteristics (working years and task and Personal Protective Equipment (PPE) use). Workers exposed to FA reported to adopt the prevention and protection measures, both collective and individual, established by the regulation in force (Legislative Decree No 81/2008).

### Formaldehyde exposure assessment

Each volunteer wore a personal radial symmetry diffusive air sampler (Radiello^®^, ICS Maugeri SpA, Pavia, Italy) (https://radiello.com/, accessed on 23 December 2021). This device was clipped to the collar to assess the air-FA concentration in the worker’s breathing zone during a standard working day. The samplers were equipped with a special sorbent cartridge containing a 35–50 Florisil mesh coated with 2,4-dinitrophenylhydrazine (DNPH). The quantification was performed by HPLC, as previously described (Squillacioti et al. 2020). As recommended by the Radiello^®^ user manual, we kept at least two cartridges of each lot as a blank. The average FA concentration over exposure time is calculated as the mass of the analyte in the cartridge over time according to the following expression: *C* = (mass of the analyte [µg]/(*Q*_k_ [ml·min^−1^]·exposure time [min]))·1,000,000. Temperature was assumed to be constantly 25 °C and results are expressed in µg·m^−3^.

### Blood sampling and SCEs assay

5–10 mL venous blood specimens for SCEs assay obtained were collected into heparinised Vacutainer^®^ (Becton, Dickinson and Company, Franklin Lakes, NJ, USA). Blood samples were coded and kept refrigerated (+ 4 °C) until analysis and processed within 2 h, as previously described. A 0.3 mL aliquot of each sample was cultured in a 25 cm^2^ flask with 6 mL of RPMI-1640 (Merck, Milan, Italy) with the addition of 20% fetal calf serum (FCS), 2% mitogenic agent phytohemagglutinin-M (Difco, 0.2 mL), l-glutamine (2 mM), antibiotics (100 μg/mL streptomycin and 100 IU/mL penicillin). Cultures were then incubated at 37 °C for 72 h, in a 5% CO_2_ atmosphere. During the last 2 h of culture 0.06 μg/mL of colchicine (Merck, Milan, Italy, 0.25 μg/mL) was added in order to arrest cells in mitosis.

During the chromosome preparation step, after a 10 min centrifugation at ≤ 800 rpm, cells were slowly resuspended in 10 mL of pre-warmed hypotonic solution (0.075 M KCl, 37 °C), and subsequently incubated in water bath 15 min at 37 °C. After a new centrifugation, cells were than fixed with a 20 min incubation in cold methanol:acetic acid (3:1) at room temperature. The fixation treatment was repeated three times. After discarding the supernatant, the remaining pellet, still dissolved in a residual fixative volume, was seeded on slides.

Bromodeoxyuridine (BrdUrd, 10 μg/mL), a thymidine analogue that during replication can be efficiently incorporated into the newly synthesized DNA strands, was added at 24 h to measure SCEs in second division metaphases. After two cell cycles in BrdUrd medium, the two sister chromatids revealed a different appearance due to the amount of BrdUrd incorporated: the lighter chromatid is the one with more BrdUrd (“bleaching” effect).

In order to allow sister chromatid differentiation, cells were thereafter stained with a 20 min incubation with 10 μg/mL fluorescence dye Hoechst 33258 (Merck, Milan, Italy) in the dark at room temperature. Cells were then irradiated 30 min with an 8-W UV lamp (254 nm) at approximately 20 cm. Subsequently, after an 1 h incubation in 2 × Standard Saline Concentration (SSC) at 60 °C, and slides were then stained with a 10 min incubation in Sörensen buffer with 5% Giemsa (Merck, Milan, Italy). Microscopic analyses were performed by a light microscope (CX40, Olympus, Tokyo, Japan) at 1000 × magnification.

The determination of the SCE/cell number for each subject was performed scoring 50 well-spread second-division metaphases containing 46 chromosomes. The replication index (RI) evaluation was performed scoring a total of 100 cells from each donor and calculated according to the following formula: RI = (M1 + 2 M2 + 3 M3)/N, where M1, M2 and M3 represent the number of cells undergoing first second and third mitosis and N is the total number of scored metaphases (NSM) (Santovito et al. 2014, 2017).

### DNA extraction and genotyping

An aliquot of all blood specimens had been stored at − 20 °C until the assay. The DNA extraction and genotypization analysis had been performed as previously described (Ruberto and Santovito 2021).

Primer sequences, melting temperatures, PCR methodologies used, and expected PCR product sizes are reported in Online Resource 1.

### Statistical analyses

The Shapiro–Wilk test and density visualization was used to test the normal distribution. The variables were described as frequency (percentage) for categorical variables, median and interquartile range (IQR) for non-normal distributed variables, and mean and standard deviation (± SD) for endpoints with normal distribution. Continuous non-normally distributed variables were compared across relevant groups using the Mann–Whitney *U* test or Wilcoxon signed-rank test and the *T*-test was used for normal-distributed ones. Fisher’s exact test served for group comparison of categorical ones. The Spearman rank correlation test was used to investigate correlations. Group tests were two-sided with *p* < 0.05 considered statistically significant.

Multivariate regression analyses were performed to calculate associations between FA exposure and genetic and clinical variables. Several regression model were created: M0 was performed to evaluate the role of exposure and smoking in the SCEs frequency alteration, 5 different models were performed in order to study the possible role of susceptibility polymorphisms, grouped by the molecular pathway they belong to (phase I (M1), phase II (M2), BER (M3), NER (M4), CK (M5), (Online Resources 2–6), in shaping the frequency of SCEs once covariated for all clinical and exposure variables. Analyses were performed using R 4.4.1 (R Project for Statistical Computing, Vienna, Austria) and SPSS Statistics (IBM SPSS Statistics, Version 27.0. Armonk, NY: IBM Corp).

## Results

The final sample includes 105 volunteers, 57 pathologists occupationally exposed to FA and 48 unexposed healthcare professionals considered as control group. The general characteristics of the subjects recruited are shown in Table 1.

**Table 1:** Demographic characteristics and air-FA exposure level of subjects belonging to the studied groups

	Pathologists (*n* = 57)	Controls (*n* = 48)	*p*-value
Sex			> 0.05
Males (%)	50.9	52.1	
Females (%)	49.1	47.9	
Age (years)			> 0.05
*Median [IQR]* *min–max*	43 [12] 25–60	38 [12] 25 – 70	
Years of employment (years)			> 0.05
*Median [IQR]* *min–max*	10 [13] 1–33	10 [5] 2–32	
Smoking habit			> 0.05
Non-smokers (%)	75.4%	81.3%	
Smokers (%)	24.6%	18.8%	
Cigarettes/die			> 0.05
*Median [IQR]* *min–max*	7.50 [12] 5–40	13 [7] 10–22	
Air-FA (μg/m^3^)			< 0.001
*Median [IQR]* *(ppm conversion)* *min–max*	55.2 [22.3] (0.045 [0.018]) 30.0–169.3	18.5 [5.4] (0.015 [0.004]) 9.2–36.3	

Continuous variables were compared by Mann–Whitney *U* test or Wilcoxon signed-rank test, while categorical variables were compared by Fisher’s exact test. *n* = number of analysed subjects

The comparison between exposed and non-exposed subjects reveals a significantly higher SCEs frequency in pathologists (*p* = 0.009), as displayed in Table 2.

**Table 2:** Comparison between exposed and control groups in terms of genotoxic outcomes

Groups	*N*	SCEs	SCEs/cell ± SD	RI ± S.D
**Pathologists**	**57**	**15,102**	5.30 ± 1.33*	1.69 ± 0.31**
Males Females Smokers Non-smokers	29 28 14 43	7877 7225 3841 11,261	5.43 ± 1.18 5.16 ± 1.49 5.49 ± 0.99 5.24 ± 1.43	1.71 ± 0.33 1.66 ± 0.28 1.82 ± 0.28^a^ 1.65 ± 0.31
**Controls**	**48**	**10,598**	**4.42 ± 1.50**	**1.92 ± 0.19**
Males Females Smokers Non-smokers	25 23 9 39	5952 4646 2566 8032	4.78 ± 1.32 4.03 ± 1.61 5.77 ± 1.13^b^ 4.11 ± 1.41	1.90 ± 0.15 1.95 ± 0.22 1.74 ± 0.15^c^ 1.97 ± 0.17

Non-normally distributed continuous variables were compared by Mann–Whitney *U* test or Wilcoxon signed-rank test, while normally distributed variables were compared by *t*-test

*N* Number of analysed subjects, *SCEs* Sister Chromatid Exchanges, *RI* (Replication Index), *S.D.* Standard Deviation

*Pathologists vs controls *p* < 0.05

**Pathologists vs controls *p* < 0.001

^a^smokers vs non-smokers *p* < 0.05 (Mann–Whitney test)

^b^smokers vs non-smokers *p* < 0.005 (*t*-test)

^c^smokers vs non-smokers *p* < 0.005 (Mann–Whitney test)

Concerning the different genotype makeup, the comparison between the *wt* and the group of who carries at least one mutated allele, reveal, in workers exposed to FA, a significantly higher SCEs frequency in the mutation carrier group for *CYP1A1* exon 7 (*p* = 0.010) and *XPC 9* (*p* = 0.040). Among controls, the *XRCC1 194 wt* group showed a higher RI compared to the mutation carrier group (*p* = 0.019).

The correlation analysis performed on the whole sample revealed that the SCEs frequency was significantly directly correlated with the age (*ρ* = 0.30, *p* < 0.01) and the smoking habit (cigarette/die and years of smoking, respectively *ρ* = 0.27, *p* < 0.01 and *ρ* = 0.28, *p* < 0.01), air-FA concentration (*ρ* = 0.29, *p* < 0.01) and negatively correlated with the RI (*r* = − 0.21, *p* < 0.05). The RI was found to be also negatively correlated with the air-FA concentration (*ρ* = − 0.39, *p* < 0.001).

M0 regression model was performed to evaluate the role of exposure and smoking in the SCEs frequency alteration. As reported in Table 3, the exposure to Air-FA influence significantly the SCEs frequency (*β* = 38.29, *p* = 0.008). Conversely, in the same model, age, sex, smoking habit and year of exposure did not show any significant role.

**Table 3:** Multivariate regression model for personal characteristics and work-related factors in predicting the SCEs frequency

	Estimate	SE	*z*	*p*	
(Intercept)	158.415	43.904	3.608	< 0.001	***
Age	1.632	1.403	1.164	> 0.05	
Sex (F)	− 10.678	14.464	− 0.738	> 0.05	
Years of Smoking	0.776	1.272	0.61	> 0.05	
Cigarette/die	0.774	1.510	0.512	> 0.05	
Years of Exposure	− 0.202	1.670	− 0.121	> 0.05	
FA-Exposure (Pathologists)	38.291	14.048	2.726	< 0.01	**

(***) < 0.001; (**) 0.001–0.01; (*) 0.01–0.05; (.) 0.05–0.1

The regression models performed separating the studied genes according to the molecular pathways they belong to (Online Resources 2–6), revealed a significant role of exposure in all models, except in M2 (phase 2 metabolic pathway). In this model, the similar variance in SCEs frequency due to the polymorphisms covered the effect of FA exposure as well, which significance remained borderline.

## Discussion

Despite the IARC classified FA as a known human carcinogen (group 1) nearly 15 years ago, the safety of workers nowadays exposed to this chemical, and particularly those employed in the anatomy pathology wards, remains a matter of concern.

Currently, although there are alternative methods to the use of FA, such as the practice of Under-Vacuum Sealing (UVS) (Bellisario et al. 2016), FA is still considered the standard fixative for routine work. In Italy, 1726 workers employed in specialised hospital activities had been estimated to be potentially exposed to FA (Scarselli et al. 2017; Dugheri et al. 2021). To safeguard workers occupationally exposed to air-FA, various limits of exposure have been proposed, even though there is no agreement between the international agencies (Dugheri et al. 2020). The current Threshold Limit Value—Time Weighted Average (TLV – TWA) recommended by the American Conference of Governmental Industrial Hygienists (ACGIH) is 120 μg/m^3^ (i.e., 0.1 ppm) and is referred to average exposure in eight-hour workdays (Dugheri et al. 2020).

Within this scenario, performing research on the various outcomes relatable to FA exposure and on other potentially influencing factors remains crucial. Specifically, the understanding of the gene-by-exposure effect could allow identifying more susceptible subjects and, eventually, defining health-based recommended limits that could be safe for all exposed workers (Faruque et al. 2020). Our aim was to deepen the possible association between exposure to air-FA and genotoxic damage. The eventual identification of significant SCEs levels according to the workers’ genetic makeup in subject exposed to a deemed safe concentration of air-FA could provide relevant information to the continuous revising process of FA exposure limits.

The median air-FA concentration measured in the exposed workers breathing zone (0.06 mg/m^3^, i.e., 0.05 ppm) is lower than most of the air-FA levels in pathology wards reported in literature. Recently, Costa et al. (2019) reported an average FA level of 0.38 ppm (i.e. 0.47 mg/m^3^) in hospital anatomy-pathology laboratories in Portugal, while Jalali et al. (2021) measured even higher concentrations (0.64 mg/m^3^) in Iranian pathology wards (Costa et al. 2019; Jalali et al. 2021). In Italy, Scarselli et al. (2017) reported a general average value of 0.49 mg/m^3^ (i.e., 0.40 ppm) for workers employed in hospital activities (Scarselli et al. 2017).

In our previous work on the same sample, we reported a significantly higher chromosomal aberration (CAs) frequency in pathologists than in controls (Ghelli et al. 2021). As well, we found a higher SCEs frequency in the exposed group, in line with previous reports (Costa et al. 2013, 2019; Santovito et al. 2014). Moreover, the RI value in pathologists was significantly lower than in the control group, contrary to Santovito et al. (2014), and negatively correlated with SCEs frequency, a further evidence of cytotoxicity induction (Santovito et al. 2014).

The association between air-FA and genotoxic damage was confirmed by correlation analysis as well, revealing that air-FA concentration was positively related to SCEs frequency and negatively to RI.

Concerning the genetic background, the comparison between *wt* and mutation carrier subjects revealed, in the pathologist group, a significantly lower SCEs frequency in workers *wt* for *CYP1A1* exon 7 (A > G) and *XPC* exon 9 (C499T). *CYP1A1* is a member of the CYP450 family playing a fundamental role in the metabolism of both endogenous and exogenous substrates, such as nutrients, drugs, and environmental carcinogens (Badal and Delgoda 2014). The enzyme coded by this gene, indeed, is mostly expressed in extra hepatic tissues where it is involved in the polycyclic aromatic hydrocarbons (PAHs) biotransformation, which is associated with lung cancer risk (Ezzeldin et al. 2019). *CYP1A1* expression is affected by environmental and genetic factors (Ezzeldin et al. 2019). Specifically, the Ile462Val polymorphism, causing a substitution in the enzyme heme-binding region, result in a twofold increase of the microsomal enzyme activity and is a risk factor for many types of cancer and hematopoietic malignancies, such as acute leukaemia (Zhuo et al. 2012; Roszak et al. 2014). However, previous studies did not report any association between this polymorphism and SCEs frequency (Carere et al. 2002; Santovito et al. 2017). The human *XPC* (*Xeroderma pigmentosum* complementation group C) gene encodes for a 940-amino acid protein essential within the NER (Nucleotide Excision Repair) pathway, involved in the early damage site recognition and DNA repair initiation (Dai et al. 2019). The abnormal expression of the XPC protein is related to cancer progression, and the TT genotype seems to be associated with an increased risk of bladder and breast cancers (Dai et al. 2019). To our knowledge, this is the first report highlighting a significant higher SCEs frequency in workers exposed to FA carrying the XPC exon 9 (C > T) polymorphism.

The regression analysis, however, did not evidence any significant influence of the studied polymorphisms in modulating the SCEs frequency, in line with literature (Costa et al. 2019). However, the FA exposure remains significant in almost all the models strengthening, once more, its role in the genotoxic damage induction. Interestingly, only few studies on occupational research topic analysed the influence of cytokine genetic polymorphisms, even though the key role of the inflammatory pathway in mediating the consequences of occupational exposures. In the present research we did not highlight any role of the studied cytokine polymorphisms in inducing SCEs, contrary to Santovito et al. (2015), which reported a role of IL-6 -174 (G > C). However, in that report, the authors specified that it is still unclear whether the genomic effects can be related to a direct action of these polymorphisms or, more likely, to the interaction with the cytokine network.

Despite evidence on the tobacco-smoking role in SCEs induction (DeMarini 2004), in our regression models, nor the number of cigarettes smoked every day, nor the years of smoking resulted significantly related with the selected outcome.

The novelty of our research consists in the combination of several different parameters. Firstly, the personal assessment of air-FA exposure provides a further element to the varied picture of FA exposure in the Italian scenario. Data concerning personal FA exposure were associated with both a DNA damage endpoint (SCEs) and the genetic makeup. The battery of genetic polymorphisms analysed belong to various biological pathways, some of them (e.g. TNF-α, IL 10, IL-6) not usually considered in relation to FA exposure because not directly conditioning the FA-metabolism pathway, but which could indirectly affect the onset of the studied outcome. This approach allowed a comparison between the exposed and the control groups and to deep the role of susceptibility biomarkers in modulating the SCEs induction in occupational studies.

The results of our study, however, should be considered carefully. Indeed, the cross-sectional design of the study did not allow making causal inferences and workers employed in pathology wards are exposed to several chemical agents other than FA that could potentially have a confounding effect in the induction of SCEs.

## Conclusion

The present study confirms once more the genotoxic effect of FA, highlighting the urgent need to put in place all the possible strategies to reduce and prevent the chronic exposure consequences, especially in working settings. Moreover, we focus our attention on the importance of susceptibility biomarkers, which could modulate the individual biological response to the xenobiotic exposure. This approach is of outmost importance in order to provide data for the definition of exposure limits that could be safe for exposed workers.

## Supplementary Information

Below is the link to the electronic supplementary material.

Supplementary file1 (DOCX 49 kb)
