# Supplementary material for: The formation of SCEs as an effect of occupational exposure to formaldehyde
Source: Arch Toxicol. 2022 Feb 12;96(4):1101–8. doi: 10.1007/s00204-022-03238-w (PMC8921006; doi:10.1007/s00204-022-03238-w)
Supplement: Supplementary file 1 — Supplementary file1 (DOCX 49 kb) [file 204_2022_3238_MOESM1_ESM.docx]

**Supplementary Information**

**The formation of SCEs as an effect of occupational exposure to formaldehyde.**

*Archives of Toxicology*

**Authors:**

Federica Ghelli^1^, Enrico Cocchi^1^, Valeria Bellisario^1^, Martina Buglisi^1^, Giulia Squillacioti^1^, Alfredo Santovito^1^ and Roberto Bono^1†^

^1^ Department of Public Health and Pediatrics, University of Turin, Via Santena 5 bis, 10126 Turin, Italy.

^2^ Department of Life Sciences and Systems Biology, University of Turin, Via Accademia Albertina 13, 10123 Turin, Italy

^†^ **Corresponding author**: [roberto.bono@unito.it](mailto:roberto.bono@unito.it)

**Online Resource 1** Primers, annealing temperatures and methodologies used for the analysed gene polymorphisms.

| **Gene** | **Sequence** | **T (°C)** | **Methodology** | **PCR product size (bp)** | **Reference** |
| --- | --- | --- | --- | --- | --- |
| *CYP1A1 exon 7 (A>G)* | 5′- AAGACCTCCCAGCGGGCAAT - 3′  5′- AAGACCTCCCAGCGGGCAAC - 3′  5′- CTCTGGTTACAGGAAGCTAT - 3′ | 60 | PCR | 162 | (Chen et al. 2001) |
| *CYP1A1*2A (T>C)* | 5’- CAGTGAAGAGGTGTAGCCGCT - 3’  5’- TAGGGAGTCTTGTCTCATGCCT - 3’ | 60 | RFLP  (MaspI) | PCR product: 340 bp  T-allele = 340 bp  C-allele = 200 and 140 bp | (Salehi et al. 2012) |
| *CYP2C19*2 (G>A)* | 5’- CAGAGCTTGGCATATTGTATC - 3’  5’- TATCGCAAGCAGTCACATAAC- 3’  5'-ACTATCATTGATTATTTCCCG-3’  5’-GTAATTTGTTATGGGTTCCT-3’ | 57 | ARMS-PCR | PCR Product: 373 bp  2*G-allele = 283 bp  2*A-allele = 129 bp. | (Bonello et al. 2010) |
| *GSTT1 (presence/absence)* | 5’- TTCCTTACTGGTCCTCACATCTC - 3’  5’- TCACCGGATCATGGCCAGCA - 3’ | 63 | PCR | 480 | (Pemble et al. 1994) |
| *GSTM1 (presence/absence)* | 5- CTGGATTGTAGCAGATCATGC - 3’  5’- CTGCCCTACTTGATTGATGGG - 3’ | 65 | PCR | 273 | (Zhong et al. 1993) |
| *GSTP1 (A>G)* | 5’- AATACCATCCTGCGTCACCT - 3’  5- TGAGGGCACAAGAAGCCCCTT - 3’ | 60 | RFLP  (BsmA I) | PCR Product: 566 bp  A-allele = 308 + 258 bp  G-allele = 258 + 219 + 89 bp | (García-González et al. 2012) |
| *XRCC1 (399, G>A)* | 5’- CAAGTACAGCCAGGTCCTAG - 3’  5’-CCTTCCCTCATCTGGAGTAC - 3’ | 60 | RFLP  (Nci I) | PCR Product: 248 bp  G-allele = 159 + 89  A-allele = 248 bp | (Matullo et al. 2001) |
| *XRCC1 (194, C>T)* | 5’- GCCCCGTCCCAGGTA – 3’  5’- AGCCCCAAGACCCTTTCACT - 3’ | 60 | RFLP  (MspI) | PCR Product: 383 bp  C-allele = 346 + 37 bp  T-allele = 383 bp | (Wang et al. 2010) |
| *XRCC1A (280, A>G)* | 5’-TGGGGCCTGGATTGCTGGGTCTG – 3’  5’- CAGCACCACTACCACACCCTGAAGG – 3’ | 60 | RFLP  (RsaI) | PCR Product: 280 bp  A-allele = 140 bp  G-allele = 280 bp | (Wang et al. 2010) |
| *XPC exon 15 (A>C)* | 5’ – ACCAGCTCTCAAGCAGAAGC – 3’  5’ – CTGCCTCAGTTTGCCTTCTC – 3’ | 60 | RFLP  (Pvu II) | PCR Product: 281 bp  A-allele = 281  C-allele = 150+131 bp | (Hu et al. 2005) |
| *XPC exon 9 (C>T)* | 5’ – TAAGGACCCAAGCTTGCCCG – 3’  5’ – CCCACTTTTCCTCCTGCTCACAG – 3’ | 60 | RFLP  (Sac II) | PCR Product: 152 bp  C-allele = 131+21 bp  T-allele = 152 bp | (Hu et al. 2005) |
| *TNF-α (-308, G>A)*   - Antisense primer - G-sense primer - A-sense primer | 5’-TCTCGGTTTCTTCTCCATCG-3’  5’-ATAGGTTTTGAGGGGCATGG-3’  5-AATAGGTTTTGAGGGGCATGA-3’ | 60 | ARMS-PCR | 184 | (Perrey et al. 1999) |
| *IL 10 -1082 (G>A)*   - Antisense primer - G-sense primer - A-sense primer | 5’-AGTGCCAACTGAGAATTTGG-3’  5’-CTACTAAGGCTTCTTTGGGAG-3’  5’-ACTACTAAGGCTTCTTTGGGAA-3’ | 60 | ARMS-PCR | 258 | (Perrey et al. 1999) |
| *IL-6 (-174, G>C)*   - Antisense primer - G-sense primer - C-sense primer | 5’-TCGTGCATGACTTCAGCTTTA-3’  5’-AATGTGACGTCCTTTAGCATG-3’  5’-AATGTGACGTCCTTTAGCATC-3’ | 60 | ARMS-PCR | 190 | (Zakharyan et al. 2012) |

**Online Resource 2**: Regression model M1, including personal and work-related characteristics and SNPs in gene coding for phase I-enzymes in predicting the SCEs frequency.

|  | **Estimate** | **SE** | **z** | **p-value** |  |
| --- | --- | --- | --- | --- | --- |
| (Intercept) | 149.668 | 44.798 | 3.341 | ~~0.001~~ < 0.005 | ** |
| Age | 1.790 | 1.407 | 1.272 | ~~0.207~~ > 0.05 |  |
| Years of employment | -0.098 | 1.691 | -0.058 | ~~0.954~~ > 0.05 |  |
| Sex (F) | -10.233 | 14.685 | -0.697 | ~~0.488~~ > 0.05 |  |
| Cigarette/die | 1.487 | 1.002 | 1.485 | ~~0.141~~ > 0.05 |  |
| Exposure (pathologists) | 37.753 | 14.404 | 2.621 | ~~0.010~~ < 0.05 | * |
| *CYP1A1 exon 7 (A>G)* (*G) | 7.926 | 14.912 | 0.532 | ~~0.596~~ > 0.05 |  |
| *CYP1A1*2A* (*C) | 0.516 | 17.317 | 0.03 | ~~0.976~~ > 0.05 |  |
| *CYP2C19*2* (*A) | -7.765 | 19.414 | -0.4 | ~~0.690~~ > 0.05 |  |

(***) < 0.001; (**) 0.001 – 0.01; (*) 0.01 – 0.05; (.) 0.05 – 0. 1.

**Online Resource 3**: Regression model M2, including personal and work-related characteristics and SNPs in gene coding for phase II-enzymes in predicting the SCEs frequency.

|  | **Estimate** | **SE** | **z** | **p-value** |  |
| --- | --- | --- | --- | --- | --- |
| (Intercept) | 137.557 | 45.609 | 3.016 | ~~0.003~~ < 0.01 | ** |
| Age | 1.809 | 1.392 | 1.3 | ~~0.197~~ > 0.05 |  |
| Years of employment | -0.052 | 1.676 | -0.031 | ~~0.975~~ > 0.05 |  |
| Sex (F) | -13.267 | 14.578 | -0.91 | ~~0.365~~ > 0.05 |  |
| Cigarette/die | 1.64 | 0.995 | 1.648 | ~~0.103~~ > 0.05 |  |
| Exposure (pathologists) | 28.610 | 15.607 | 1.833 | ~~0.070~~ > 0.05 | . |
| *GSTT1* (*GSTT1+*) | 4.761 | 14.806 | 0.322 | ~~0.749~~ > 0.05 |  |
| *GSTM1* (*GSTM1+*) | 19.866 | 15.227 | 1.305 | ~~0.195~~ > 0.05 |  |
| *GSTP1* (*G) | 10.220 | 14.703 | 0.695 | ~~0.489~~ > 0.05 |  |

(***) < 0.001; (**) 0.001 – 0.01; (*) 0.01 – 0.05; (.) 0.05 – 0. 1.

**Online Resource 4**: Regression model M3, including personal and work-related characteristics and SNPs in gene coding for BER-pathway enzymes in predicting the SCEs frequency.

|  | **Estimate** | **SE** | **z** | **p-value** |  |
| --- | --- | --- | --- | --- | --- |
| (Intercept) | 145.493 | 45.173 | 3.221 | ~~0.002~~ < 0.01 | ** |
| Age | 1.987 | 1.428 | 1.391 | ~~0.167~~ > 0.05 |  |
| Years of employment | -0.238 | 1.734 | -0.137 | ~~0.891~~ > 0.05 |  |
| Sex (F) | -10.398 | 14.615 | -0.711 | ~~0.479~~ > 0.05 |  |
| Cigarette/die | 1.651 | 1.047 | 1.578 | ~~0.118~~ > 0.05 |  |
| Exposure (pathologists) | 40.785 | 14.181 | 2.876 | ~~0.005~~ < 0.01 | ** |
| *XRCC1 (399, G>A)* (*A) | 0.790 | 14.463 | 0.055 | ~~0.957~~ > 0.05 |  |
| *XRCC1 (194, C>T)* (*T) | -14.525 | 15.497 | -0.937 | ~~0.351~~ > 0.05 |  |
| *XRCC1A (280, A>G)* (*G) | 12.040 | 20.456 | 0.589 | ~~0.558~~ > 0.05 |  |

(***) < 0.001; (**) 0.001 – 0.01; (*) 0.01 – 0.05; (.) 0.05 – 0. 1.

**Online Resource 5**: Regression model M4, including personal and work-related characteristics and SNPs in gene coding for NER-pathway enzymes in predicting the SCEs frequency.

|  | **Estimate** | **SE** | **z** | **p-value** |  |
| --- | --- | --- | --- | --- | --- |
| (Intercept) | 138.119 | 45.089 | 3.063 | ~~0.003~~ < 0.01 | ** |
| Age | 1.964 | 1.395 | 1.408 | ~~0.162~~ > 0.05 |  |
| Years of employment | -0.395 | 1.672 | -0.236 | ~~0.814~~ > 0.05 |  |
| Sex (F) | -12.767 | 14.323 | -0.891 | ~~0.375~~ > 0.05 |  |
| Cigarette/die | 1.253 | 1 | 1.253 | ~~0.214~~ > 0.05 |  |
| Exposure (pathologists) | 37.786 | 13.976 | 2.704 | ~~0.008~~ < 0.01 | ** |
| *XPC exon 15 (A>C)* (*C) | 13.858 | 13.821 | 1.003 | ~~0.319~~ > 0.05 |  |
| *XPC exon 9 (C>T)* (*T) | 14.659 | 16.925 | 0.866 | ~~0.389~~ > 0.05 |  |

(***) < 0.001; (**) 0.001 – 0.01; (*) 0.01 – 0.05; (.) 0.05 – 0. 1.

**Online Resource 6**: Regression model M5, including personal and work-related characteristics and SNPs in gene involved in cytokine secretion in predicting the SCEs frequency.

|  | **Estimate** | **SE** | **z** | **p-value** |  |
| --- | --- | --- | --- | --- | --- |
| (Intercept) | 158.824 | 44.367 | 3.58 | ~~0.001~~ < 0.001 | *** |
| Age | 1.672 | 1.411 | 1.185 | ~~0.239~~ > 0.05 |  |
| Years of employment | -0.136 | 1.691 | -0.081 | ~~0.936~~ > 0.05 |  |
| Sex (F) | -13.511 | 14.952 | -0.904 | ~~0.368~~ > 0.05 |  |
| Cigarette/die | 1.556 | 1.087 | 1.432 | ~~0.155~~ > 0.05 |  |
| Exposure (pathologists) | 37.870 | 14.242 | 2.659 | ~~0.009~~ < 0.05 | ** |
| *TNF-α (-308, G>A)* (*A) | -2.563 | 13.898 | -0.184 | ~~0.854~~ > 0.05 |  |
| *IL 10 -1082 (G>A)* (*A) | 7.439 | 14.232 | 0.523 | ~~0.602~~ > 0.05 |  |
| *IL-6 (-174, G>C)* (*C) | -7.095 | 16.004 | -0.443 | ~~0.659~~ > 0.05 |  |

(***) < 0.001; (**) 0.001 – 0.01; (*) 0.01 – 0.05; (.) 0.05 – 0. 1.

**References**

Bonello L, Armero S, Ait Mokhtar O, et al (2010) Clopidogrel Loading Dose Adjustment According to Platelet Reactivity Monitoring in Patients Carrying the 2C19*2Loss of Function Polymorphism. J Am Coll Cardiol 56:1630–1636. https://doi.org/https://doi.org/10.1016/j.jacc.2010.07.004

Chen S, Xue K, Xu L, et al (2001) Polymorphisms of the CYP1A1 and GSTM1 genes in relation to individual susceptibility to lung carcinoma in Chinese population. Mutat Res - Mutat Res Genomics 458:41–47. https://doi.org/10.1016/S1383-5726(01)00011-5

García-González MA, Quintero E, Bujanda L, et al (2012) Relevance of GSTM1, GSTT1, and GSTP1 gene polymorphisms to gastric cancer susceptibility and phenotype. Mutagenesis 27:771–777. https://doi.org/10.1093/mutage/ges049

Hu Z, Wang Y, Wang X, et al (2005) DNA repair gene XPC genotypes/haplotypes and risk of lung cancer in a Chinese population. Int J Cancer 115:478–483. https://doi.org/10.1002/ijc.20911

Matullo G, Palli D, Peluso M, et al (2001) XRCC1, XRCC3, XPD gene polymorphisms, smoking and 32P-DNA adducts in a sample of healthy subjects. Carcinogenesis 22:1437–1445. https://doi.org/10.1093/carcin/22.9.1437

Pemble S, Schroeder KR, Spencer SR, et al (1994) Human glutathione S-transferase Theta (GSTT1): cDNA cloning and the characterization of a genetic polymorphism. Biochem J 300:271–276. https://doi.org/10.1042/bj3000271

Perrey C, Turner SJ, Pravica V, et al (1999) ARMS-PCR methodologies to determine IL-10, TNF-α, TNF-β and TGF-β1 gene polymorphisms [2]. Transpl. Immunol. 7:127–128

Salehi Z, Gholizadeh L, Vaziri H, Madani AH (2012) Analysis of GSTM1, GSTT1, and CYP1A1 in Idiopathic Male Infertility. Reprod Sci 19:81–85. https://doi.org/10.1177/1933719111413302

Wang Q, Sun Y, Qiu Y-L, et al (2010) Genetic polymorphisms of XRCC1, HOGG1 and MGMT and micronucleus occurrence in Chinese vinyl chloride-exposed workers. Carcinogenesis 31:1068–1073. https://doi.org/10.1093/carcin/bgq075

Zakharyan R, Petrek M, Arakelyan A, et al (2012) Interleukin-6 promoter polymorphism and plasma levels in patients with schizophrenia. Tissue Antigens 80:136–142. https://doi.org/10.1111/j.1399-0039.2012.01886.x

Zhong S, Wyllie AH, Barnes D, et al (1993) Relationship between the gstm1 genetic polymorphism and susceptibility to bladder, breast and colon cancer. Carcinogenesis 14:1821–1824. https://doi.org/10.1093/carcin/14.9.1821
